# Nocturnal Continuous Positive Airway Pressure (nCPAP) Decreases High-Sensitivity C-Reactive Protein (hs-CRP) in Obstructive Sleep Apnea-Hypopnea Syndrome

**DOI:** 10.1155/2020/8913247

**Published:** 2020-11-01

**Authors:** Sameh Msaad, Akram Chaabouni, Rim Marrakchi, Mariem Boudaya, Amina Kotti, Walid Feki, Kamel Jamoussi, Samy Kammoun

**Affiliations:** ^1^Department of Respiratory and Sleep Medicine, Hédi Chaker University Hospital of Sfax, Tunisia; ^2^University of Sfax, Faculty of Medicine of Sfax, Tunisia; ^3^Department of Biochemistry, Hédi Chaker University Hospital of Sfax, Tunisia

## Abstract

**Background:**

Systemic and airway inflammation has recently been linked to obstructive sleep apnea-hypopnea syndrome (OSAHS) and is considered to be a probable risk factor for OSAHS-induced cardiovascular damage. High-sensitivity C-reactive protein (hs-CRP), as an inflammatory mediator, may be useful for the prediction of the risk of cardiovascular disease (CVD) and assessment of nocturnal continuous positive airway pressure (nCPAP) therapy effect in OSAHS patients.

**Methods:**

A prospective, controlled, cross-sectional study was conducted on 64 consecutive adult subjects with suspected sleep-disordered breathing (SDB).

**Results:**

OSAHS was confirmed in 43 patients (24 normotensive and 19 hypertensive patients) and ruled out in 21 normotensive subjects (controls). The median plasma level of hs-CRP did not differ significantly between OSAHS patients and controls. It showed an unmarked rise with the severity of OSAHS (*p* = 0.20) and was not correlated with AHI (*p* = 0.067; *r* = 0.28). After adjusting for cervical perimeter (CP), waist-to-hip ratio (WHR), and blood sugar level, hs-CRP level of 1 mg/dL or greater was significantly more often observed in OSAHS patients compared with controls (*p* = 0.032; OR = 5.60) and was also significantly associated with AHI (*p* = 0.021). A significant decrease in the median plasma hs-CRP level was observed in CPAP compliant patients (*p* = 0.006). Of those, only normotensive patients showed a significant decrease in plasma hs-CRP level. In hypertensive ones, however, the hs-CRP level dropped but not significantly. Using a linear regression model, the change in hs-CRP level (*Δ*hs-CRP) following a 6-month-nCPAP therapy was found to positively correlate with the baseline hs-CRP level for both hypertensive (*p* = 0.02; *r* = 0.68), and even more normotensive OSAHS patients (*p* < 0.0001; *r* = 0.89).

**Conclusion:**

nCPAP therapy may have a cardiovascular protective effect in OSAHS patients. hs-CRP level would be useful as a valuable predictor of success in OSAHS treatment monitoring.

## 1. Introduction

Obstructive sleep apnea syndrome (OSAHS) is a very common sleep-disordered breathing (SDB) condition, with reported rates of 3-7% in the general adult population [1]. It is caused by recurrent events of partial or complete collapse of the pharynx during sleep leading to hypopnea and apnea, respectively [1]. As apnea and hypopnea usually resolve with a sudden airway opening, an arousal and/or transient nocturnal desaturation, loud snoring, sleep disruption, and repetitive episodes of brief desaturation/reoxygenation sequences are commonly observed in patients with OSAHS.

Several clinical outcomes including excessive daytime sleepiness, cognitive dysfunction, concentration difficulties, respiratory complications, metabolic abnormalities, and cardiovascular diseases (CVD) are associated with untreated OSAHS. Among these multiple issues, CVD such as high blood pressure, heart failure, arrhythmias, coronary artery insufficiency, and cerebrovascular stroke are the most common, as was shown by several epidemiological studies [2–5]. Therefore, OSAHS was suggested as a probable risk factor for cardiovascular morbidity and mortality [5]. Oxidative stress, systemic inflammatory responses, hypercoagulation, and sympathetic nervous activation triggered by apnea-induced intermittent hypoxemia and sleep fragmentation might cause endothelial dysfunction and contribute to the development and progression of atherosclerosis in untreated OSAHS patients [4, 6, 7]. However, evidence establishing underlying physiopathological mechanisms of this relationship is not yet available [8].

Several molecules have been studied as possible biomarkers in the plausible relationship between OSAHS and CVD, one of the most widely studied being the C-reactive protein (CRP). CRP is an acute-phase protein that is mainly produced in the liver by IL-6 as part of an inflammatory response. It is thought to participate actively in the development of atheromatous lesions by inducing and enhancing the expression of adhesion molecules [9]. Compared to other biomarkers, its most important advantage is to have stable serum levels over long periods of time without diurnal variation, and to be easily, rapidly, and economically measured in blood samples with available high-sensitivity assays (hs-CRP). Increased serum level of hs-CRP is known as a sensitive systemic marker of inflammation and tissue injury [10]. Therefore, it was investigated as a biomarker for cardiac damage in apparently healthy subjects [11, 12], as well as in patients with OSAHS. hs-CRP evolution pattern in OSAHS patients treated with CPAP has also been assessed. However, previously reported results in this regard were conflicting. An independent association between OSAHS and hs-CRP has been suggested by a few population-based studies that have shown increased serum levels of hs-CRP in patients with OSAHS [13, 14], but not all studies have been able to replicate those results [15]. No conclusion could yet be reached because of the small sample size of reported studies and inadequate consideration of confounding factors. Yet, it is well known that hs-CRP level is strongly influenced by confounders such as gender, age, body mass index (BMI), creatinine clearance, and comorbidities [15, 16]. In other investigations, substantial reductions in serum levels of hs-CRP under effective CPAP therapy were not regularly reported [17–19], and the value of hs-CRP level as a therapeutic target in patients with OSAHS remains considerably controverted.

The objective of the present study was to investigate the potential causal role of OSAHS in CVD, through an examination of the association between OSAHS and serum levels of hs-CRP, independent of potential confounding variables. The impact of a 6-month-CPAP therapy in hs-CRP was also evaluated in order to know if CPAP therapy had an impact on cardiovascular outcomes in patients with OSAHS, and whether hs-CRP levels could be useful as a valuable predictor in OSHAS treatment monitoring.

## 2. Methods

### 2.1. Study Design

A prospective, controlled, cross-sectional study was conducted over the period 2013 to 2015. Ethics approval for the study was obtained, as well as informed consent from each patient enrolled in the study.

### 2.2. Subjects

The study subjects were recruited among adults aged 18 years and older who were referred to the sleep disorders unit at the Department of Pulmonology and Allergy for suspected sleep-related breathing disorders (SRBD).

Subjects with a previously known history of cardiovascular conditions including uncontrolled systemic hypertension, coronary artery disease, congestive heart failure, cardiac rhythm disorders, and stroke were considered ineligible. Patients with unknown cardiovascular conditions were not included either if initial assessment revealed any cardiovascular symptom or sign such as orthopnea, dyspnea on exertion, or leg edema or pathological electrocardiogram. However, systematic echocardiographic assessment was not possible because of lack of means. Other exclusion criteria included refusal to take part in the study, a preceding diagnosis of SRDB, central sleep apnea-hypopnea syndrome or Cheyne-Stokes respiration more than 10% of the total recording time on polygraphy, major psychiatric disorders, severe lung diseases, diabetes mellitus, dyslipidemia, dysthyroidism, inflammatory and malignant diseases, pregnancy, long-term use of sedative-hypnotic and/or muscle relaxant medications, and a recent history of trauma or operation (less than 6 months).

### 2.3. Study Protocol

Among a total of 456 patients referred for SRBD over the study period, only 64 subjects could be enrolled. Clinical assessment, nocturnal polygraph recording, and blood collections were performed for each included subject.

#### 2.3.1. Clinical Data

Clinical data were collected on the first office visit. Demographic parameters including age and gender, smoking status, and personal pathological antecedents were also determined. The Arabic version of the Epworth sleepiness score (ESS) was used to estimate the severity of daytime sleepiness. An ESS of 11 or higher was considered as an indicator of excessive daytime sleepiness. The BMI was calculated by dividing the subject's weight (in kilograms) by the square of his/her height (in centimeters). Daytime blood pressure (BP) was determined based on the average of at least three-seated measurements automatically carried out at 1 to 2-minute intervals in the right upper arm, with the subject at rest for at least 10 minutes. Subjects were defined as normotensive if systolic blood pressure (SBP) was ≤139 mmHg, and their diastolic blood pressure (DBP) was ≤89 mmHg, with no history of hypertension. Subjects were classified as hypertensive if they had a history of confirmed hypertension or when SBP was ≥140 mmHg and/or DBP ≥ 90 mmHg.

#### 2.3.2. Nocturnal Polygraph Recording

An overnight type III polysomnography (PSG) (Embla™ N7000, ResMed Corp., San Diego, CA, United States) was performed in the sleep unit for all patients. PSG consisted in the monitoring of the following variables including nocturnal oxygen saturation and cardiac pulse rate by transcutaneous oximetry, nasal pressure using nasal prongs, snoring by a contact microphone placed on the anterior neck, as well as thoracic and abdominal respiratory movements using respiratory inductance plethysmography. Scoring of respiratory events was carried out by one doctor according to the 2012 American Association of Sleep Medicine (AASM) manual for the scoring of sleep and associated events. Therefore, apnea was scored in the presence of a ≥90% decrease in airflow amplitude relative to the baseline for 10 seconds or longer, with continuous thoracic and abdominal movement. Hypopnea was defined as an event of at least 10 seconds characterized by a 30% decrease in the airflow amplitude relative to the baseline with an associated oxygen desaturation of 3% or more. Subjects without any evocative symptoms apart from snoring and AHI < 5 were classified as controls, while symptomatic patients with AHI ≥ 5/hour were diagnosed as having OSAHS. According to the recorded AHI, OAHS was graded as mild (5/h ≤ AHI < 15/h), moderate (15/h ≤ AHI < 30/h), or severe (AHI ≥ 30/h).

#### 2.3.3. Blood Collection and Analysis

At baseline, morning (8 am) fasting blood samples were drawn using standard venepuncture from each participant enrolled in the study. New blood samples during a scheduled morning appointment were collected after the first 6 months of nCPAP therapy for those who were treated by nCPAP, or at the end of a six-month follow-up period for those who received no treatment. Lithium heparin tubes were used for blood glucose, serum creatinine, blood urea nitrogen (BUN), and hs-CRP, while EDTA tubes were used for glycated hemoglobin (HbA1c). hs-CRP was measured by nephelometric technology on the BN ProSpec System, a fully automated bench top analyzer. The lower limit of detection of hs-CRP levels was 0.18 mg/L. The assay range was between 0.18 and 50 mg/l. The reproducibility value was 6.8% at 1.16 mg/l. The calibration stability was estimated to be around 60 days [20].

#### 2.3.4. Other Examinations

Electrocardiograms, chest X-rays, and spirometry tests were systematically performed for each enrolled participant in order to ensure that all eligibility criteria were met. Respiratory function parameters included forced vital capacity (FVC), forced expiratory volume in 1 second (FEV1), both expressed as absolute value in litres and FEV1/FVC ratio.

#### 2.3.5. CPAP Therapy and Follow-Up

A constant flow nCPAP therapy after a short in-hospital auto-titration was provided for all severe OSAH patients via the ResMed S9 Elite CPAP machine (ResMed Ltd Bella Vita, NSW, Australia). Control visits were conducted 1, 3, and 6 months after the nCPAP therapy initiation. At each appointment, compliance with the nCPAP therapy was assessed by the device card data. The median nightly nCPAP use was defined as the total recorded hours in the device timer divided by the number of nights of use between therapy initiation and follow-up control. A median use of at least 4 hours per night and for >5 days a week was accepted as the only criterion of good compliance [21].

### 2.4. Statistical Analysis

A Statistical Package for the Social Sciences (SPSS for Windows, version 20.0, Chicago, IL, USA) was used for all analyses. Shapiro-Wilk test was performed to check the data for normality. Descriptive data with normal distribution were expressed as mean and standard deviation (SD), while those not following normal distribution were expressed as median and minimum and maximum values.

According the sampling distribution, one-way analysis of variance (ANOVA), the Kruskal-Wallis test for continuous variables, and the chi-square test for dichotomous variables were performed to compare groups. The Wilcoxon test was used in each study group to compare hs-CRP levels at baseline and after 6-month-nCPAP therapy for OSAHS patients treated with nCPAP or after a 6-follow-up period for untreated participants. Multivariate analysis in logistic regression was performed in order to explore the correlation between serum levels of hs-CRP with clinical, biochemistry, and polygraphic variables. *p* < 0.05 was considered statistically significant.

## 3. Results

### 3.1. Demographic and Sleep Characteristics of the Study Sample

**Figure 1:**
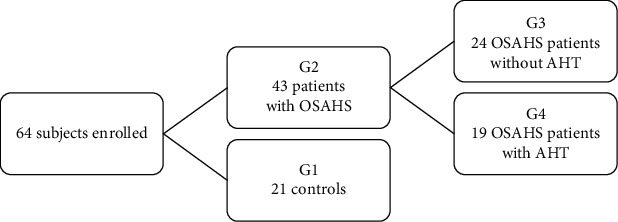
Composition of the study population. G: group; OSAHS: obstructive sleep apnea-hypopnea syndrome. AHT: arterial hypertension.

Sixty-four (64) consecutive subjects (age = 44.4 ± 10.9 − year − old, 32 males/32 females, BMI = 32.9 ± 5.9 kg/m^2^) were enrolled in the study. Forty-three (43) patients had OSAHS (OSAHS Group G2 with AHI = 28 ± 16.95/h), and 21 subjects showed no OSAHS (control group G1, AHI = 2.6 ± 1.27). Of the 43 OSAHS patients, 10 (23.3%) had an AHI of 5-14.9 (mild OSAHS), 16 (37.2%) had an AHI of 15-29.9 (moderate OSAHS), 17 (39.5%) had an AHI of 30 or more (severe OSAHS), whereas hypertension was found in 24 patients (Group G4, 55.81%), versus 19 patients without hypertension (Group G3, 44.18%) (Figure 1).

Regarding demographic characteristics (age, gender distribution, BMI, and smoking status) and BP status, no significant difference was found between OSAHS and control groups (G1 vs. G2). Demographic characteristics were also comparable between all study groups (G1, G2, G3, and G4), except for the BMI that was significantly greater in hypertensive OSAHS patients compared to normotensive OSAHS patients (G4 vs. G3; *p* = 0.032). A significantly higher ESS was found in OSAHS patients in comparison with controls (*p* = 0.013), as well as in hypertensive OSHAS patients as compared with normotensive OSHAS patients (*p* = 0.015). The greatest AHI was seen in hypertensive OAHS patients (AHI = 29.7 ± 16). The median of oxygen desaturation index (ODI) in the OAHS group was significantly higher than that in the control group (*p* = 0.0001), but was similar in OSAHS patients with and without hypertension. Comparison of serum biochemistry parameters in all groups showed no significant difference except for the fasting blood glucose level that was significantly higher in the OSAHS patients compared with controls (*p* = 0.029). Spirometric values (FEV1, FVC, and FEV1/FVC) did not differ among the four study groups. General characteristics of the four study groups are summarized in Table 1.

### 3.2. Evaluation of Baseline Serum Levels of hs-CRP

**Figure 2:**
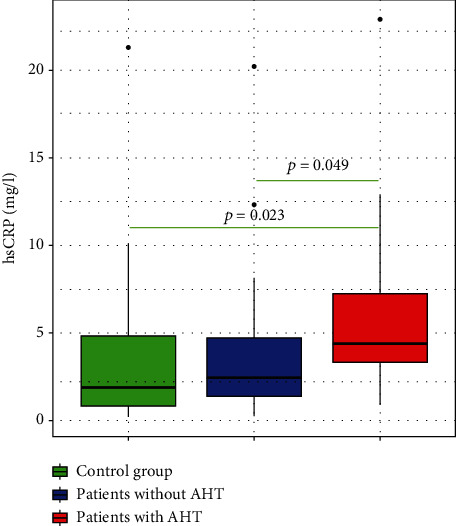
Comparison of the plasma levels of hs-CRP in controls, hypertensive, and normotensive OSAHS patients. hs-CRP: high-sensitivity C-reactive protein. OSAHS: obstructive sleep apnea-hypopnea syndrome.

The baseline serum levels of hs-CRP ranged from 0.19 to 22.90 mg/L, with a median value of 3.30 mg/L. Although OSAHS patients showed slightly higher levels of hs-CRP (3.8 [0.24-22.90]) than controls (1.86 [0.19-21.3]), the difference between the two groups was not significant. In addition, the baseline serum level of hs-CRP showed a slight rise with the severity of OSAHS (*p* = 0.20) and was not correlated with AHI (*p* = 0.067; *r* = 0.28). A significantly higher hs-CRP level was found in hypertensive OSAHS patients in comparison with normotensive OSAHS patients (respectively, 4.61 [0.87-22.90] et 2.60 mg/L [0.24-20.20]; *p* = 0.049) and controls (*p* = 0.023) (Figure 2).

A baseline serum hs-CRP level of 1 mg/L or greater was significantly more often observed in OSAHS patients compared with controls (42.9% vs. 9.3%, respectively; *p* = 0.003) (Table 2).

This association remained statistically significant after adjusting for the three variables that were found to be significantly different between the two groups, including CP, WHR, and fasting blood glucose level (*p* = 0.032; OR = 5.60). In univariate analysis of several clinical, biochemical, spirometric, and polygraphic variables on patients with OAHS, only AHI was significantly associated with a baseline serum hs-CRP level of 1 mg/L or greater (*p* = 0.021) (Table 3).

### 3.3. Effect of nCPAP Therapy

Of 27 OSAHS patients treated with nCPAP, 23 (12 normotensive and 11 hypertensive) were considered compliant while 4 were noncompliant, therefore excluded from the nCPAP trial. During the study period, no significant change was observed in the BMI of both hypertensive and normotensive nCPAP compliant patients with OSAHS as well as of controls.

**Figure 3:**
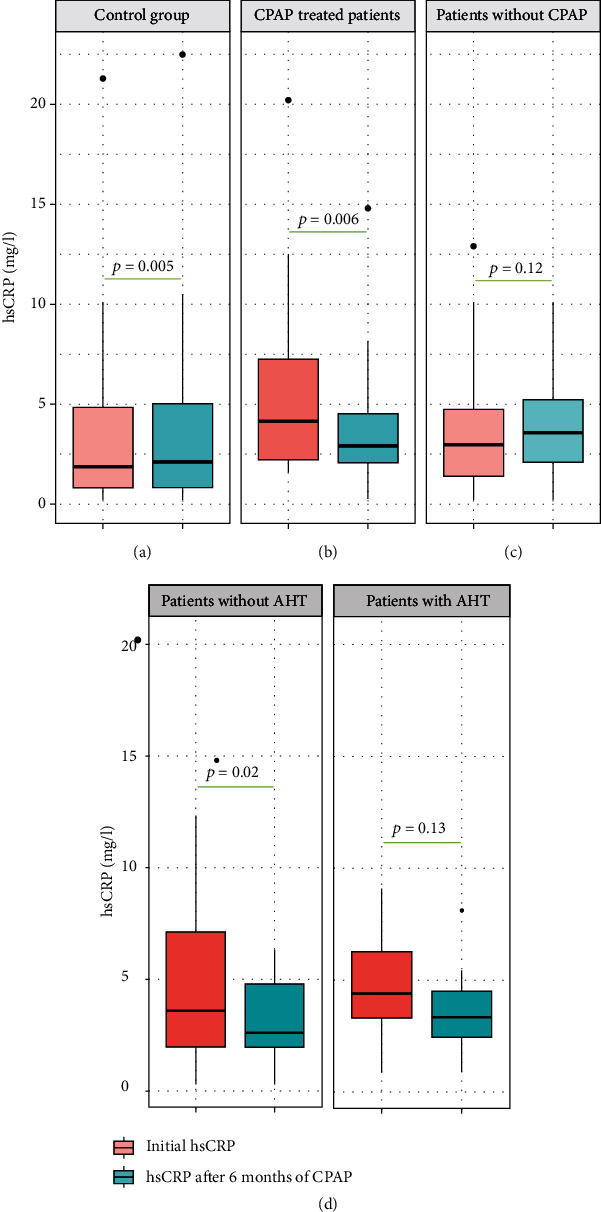
(a–d) Effects of 6 months of nCPAP therapy on the plasma hs-CRP levels. nCPAP: nocturnal continuous positive airway pressure. hs-CRP: high-sensitivity C-reactive protein. OSAHS: obstructive sleep apnea-hypopnea syndrome. AHT: arterial hypertension.

A significant decrease in serum levels of hs-CRP was reported in nCPAP-compliant patients (from 4.11 [0.24-20.20] to 2.90 [0.24-14.8]; *p* = 0.006). Of those, only normotensive patients (*n* = 12) showed a significant decrease in serum levels of hs-CRP (from 3.57 [0.24-20.20] to 2.60; [0.24-14.80]; *p* = 0.02), while in hypertensive ones (*n* = 11), hs-CRP level dropped but not significantly (from 4.39 [0.87-9.10] to 3.31; [0.91-8.12]; *p* = 0.13). In contrast, a slight increase was observed in both controls and untreated OSAHS patients (from 2.96; [0,45-12.9] to 3.55; [0.79-8.50]; *p* = 0.12) (Figure 3).

**Figure 4:**
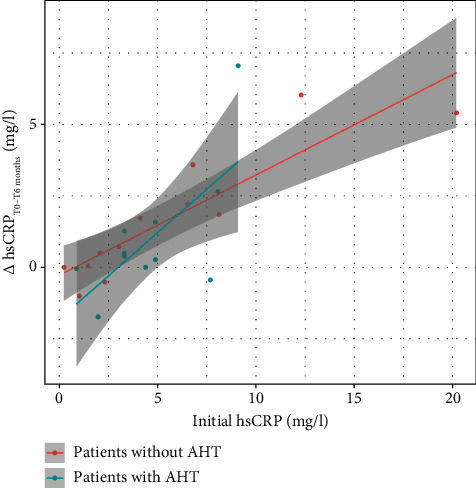
Linear regression model showing the correlation of the amplitude of variations in hs-CRP serum levels after a 6-month-nCPAP therapy (*Δ*hs-CRP [T0-T6]) with baseline levels of serum hs-CRP for hypertensive and normotensive OSAHS patients. In both groups, (*Δ*hs-CRP [T0-T6]) were more marked in patients with higher baseline hs-CRP serum levels. hs-CRP: high-sensitivity C-reactive protein. nCPAP: nocturnal continuous positive airway pressure. OSAHS: obstructive sleep apnea-hypopnea syndrome. AHT: arterial hypertension. *Δ*hs-CRP [T0-T6-month-CPAP therapy).

Using a linear regression model (Figure 4), the change in serum level of hs-CRP (*Δ*hs-CRP) following a 6 month-nCPAP therapy was found to be positively correlated with the baseline hs-CRP level for both normotensive OSAHS patients (*p* < 0.0001; *r* = 0.89) and hypertensive ones (*p* = 0.02; *r* = 0.68). No significant correlation was found with ESS (*r* = −0.20; *p* = 0.35), AHI (*r* = 0.17; *p* = 0.42), mean transcutaneous oxygen saturation (SpO_2_) (*r* = 0.001; *p* = 0.99), min SpO_2_ (*r* = 0.24; *p* = 0.25), and total sleep time with SpO_2_ < 90% (TST90%) (*r* = 0.18; *p* = 0.41).

## 4. Discussion

The present study has two main findings. First, in the absence of confounding demographic and medical conditions, OSAHS was not associated with increased serum levels of hs-CRP. Second, a 6-month-nCPAP therapy reduced significantly the serum levels of hs-CRP in compliant OSAHS patients without AHT. A dose-response relationship between the baseline serum level of hs-CRP and the amplitude of its variation after a 6-month-nCPAP therapy was also observed.

### 4.1. The Relationship between Serum Levels of hs-CRP and OSAHS

In this study, the median serum level of hs-CRP did not differ significantly between OSAHS patients and controls. It showed an insignificant rise with the severity of OSAHS (*p* = 0.20) and was not correlated with AHI (*p* = 0.067; *r* = 0.28). After adjusting for CP, WHR, and fasting serum glucose level, a hs-CRP level of 1 mg/dL or greater was significantly more often noted in OSAHS patients compared with controls (*p* = 0.032; OR = 5.60) and was also significantly associated with AHI (*p* = 0.021).

hs-CRP is a highly sensitive biomarker of systemic inflammation, infection, and tissue damage. In consideration of the important role that inflammatory processes play in the physiopathology of CVD and atherosclerosis, CRP, as an inflammatory biomarker, had been suggested to be a marker for atherosclerosis and cardiovascular risk prediction [12, 22]. The Center for Disease Control and Prevention (CDC) and the American Heart Association (AHA) jointly issued a consensus statement that identified hs-CRP as the best inflammatory biomarker to predict cardiovascular risk in primary prevention. According to the CDC and AHA statement, a low risk for CVD is defined as hs − CRP < 1 mg/L, average risk as 1 to 3 mg/L, and high risk as >3 mg/L [23]. In the literature, several researches revealed the presence of CRP in atherosclerotic plaques, more specifically in the vascular intima, which suggests a possible role of increased levels of CRP in endothelial dysfunction and atherosclerosis pathogenesis [13, 24]. Thus, hs-CRP has been suggested as a mediator for cardiovascular damage.

Intermittent hypoxemia in OSAHS has been shown to stimulate inflammatory responses which results in increased hepatic production of CRP [25–27]. Therefore, the raised hs-CRP level has been suggested to be associated with OSAHS and proposed as a useful biomarker for OSAHS-induced-cardiovascular damage. Nonetheless, the relationship between OSAHS and hs-CRP remains under debate, on account of the impact of confounding factors, such as obesity and diabetes. Besides, the results so far published are inconsistent. Our findings were concordant with those of some studies in which the hs-CRP levels detected in OSAHS patients were comparable to controls and had no significant association with the severity of OSAHS. Barcelo et al. [28] found that CRP levels in nonobese OSAHS patients (*n* = 24) did not differ significantly from nonobese controls (*n* = 18). As for Ryan et al. [29], they showed in a study including 30 controls, 35 OSAHS patients with AHI < 30/h, and 31 OSAHS patients with AHI ≥ 30/h that CRP levels were not associated with the severity of OSAHS (*p* = 0.289).

In contrast to these findings, Kokturk et al. [30] reported that hs-CRP levels were significantly higher in 94 OSAHS patients as compared with 57 controls matched for age, sex-ratio, and conventional risk factors for CVD (3.36 ± 6.5 mg/L vs. 1.16 ± 5.8 mg/L; *p* < 0.05). The hs-CRP levels were also shown to positively correlate with AHI in OSAHS patients (*r* = 0.61; *p* < 0.001). In a meta-analysis by Li et al. [10], involving 552 subjects (360 OSAHS patients and 192 healthy control subjects), serum hs-CRP levels in the OSAHS group were 1.57  mmol/L higher than that in the control group (95% confidence interval: 0.96–2.18, *p* < 0.01). Those with higher BMI and AHI showed greater differences in CRP/hs-CRP levels. In our study, a hs-CRP level ≥ 1 mg/dL was significantly more often observed in OSAHS patients compared with controls (*p* = 0.032; OR = 5.60) and was also significantly associated with AHI (*p* = 0.021). However, these results should be treated with caution because of the small sample size in the control group (4 subjects). In another study, to further examine the physiopathological role of CRP in the atherosclerosis process, Minoguchi et al. [26] measured plasma CRP levels and carotid intima-media thickness (CIMT) using ultrasound in 36 OSAHS patients and 16 control subjects. Results revealed significantly increased plasma CRP levels (*p* < 0.003) and CIMT (*p* < 0.001) in OSAHS patients compared with those in the control group. Accordingly, high hs-CRP levels were found to be significantly associated with increased CIMT as was reported by Kurkowska et al. [22].

Based on these findings, the relationship between OSAHS, the increased hs-CRP levels, and OSAH-induced CVD could be better understood. Actually, it has been demonstrated that chronic intermittent hypoxemia in OSAHS induces enhanced sympathetic activity with oxidative stress, both of which are thought to contribute to the development of chronic systemic inflammation with an increased level of inflammatory biomarkers, especially hs-CRP [31]. Taking into account that inflammation plays an essential role in the pathophysiologic processes leading to atherosclerosis and CVD, OSAHS as it is responsible for chronic systemic inflammation has been suggested as a potential CVD risk factor. Similarly, hs-CRP has been suggested as both a marker and a mediator for cardiovascular damage resulting from OSAHS.

In our study, we found that the hs-CRP levels were significantly higher in hypertensive OSAHS patients compared with normotensive ones ((4.61 mg/L [0.87-22.90]) vs. (2.60 mg/L [0.24-20.20]), respectively; *p* = 0.049). Similarly, Qian et al. [32] found significantly increased hs-CRP levels in hypertensive OSAHS patients compared with normotensive ones (4 ± 2.2 mg/L vs. 2.8 ± 1.1 mg/L; *p* < 0.05). Hypertension causes increased release of both ICAM-1 and IL-6; that in turn stimulate CRP release [33].

### 4.2. Effect of nCPAP on the Serum Levels of hs-CRP

Three major findings in our study regarding the effect of a 6 month-nCPAP therapy. First, in comparison to control subjects, a significant decrease in serum levels of hs-CRP was observed in compliant OSAHS patients. Second, among those, the decrease of serum levels of hs-CRP was significant in normotensive patients, but not in hypertensive ones. Third, the effect of the nCPAP therapy was more marked in OSAHS patients with higher baseline serum levels of hs-CRP whether they were normotensive or hypertensive. However, this effect was neither correlated with ESS nor with the polygraph indicators (AHI, mean SpO_2_, min SPO_2_, and TST90%).

Our findings are consistent with some previous studies. Yokoe et al. [14] reported that 30 patients with moderate and severe OSAHS showed a significant decrease in hs-CRP levels (from 2.9 ± 0.2 mg/L to 1.1 ± 0.3 mg/L; *p* < 0.0001), after just one month of CPAP therapy. In a meta-analysis by Guo et al. [17], including 1199 patients, the application of 3 months of CPAP therapy was found to result in a significant fall in the serum hs-CRP levels (*p* < 0.001). Schiza et al. [34] assessed CRP levels in 528 patients with OSAHS under CPAP therapy. They found that after 6 months of good compliance to CPAP, CRP levels decreased from 7.4 ± 6.2 mg/L to 3.1 ± 2.9 mg/L (*p* < 0.001), while BMI levels remained fairly stable. As far as Ishida et al. [18] are concerned, they showed that the magnitude of reduction in the CRP levels in 55 OSAHS patients after 6 months of good compliance to CPAP was observed in those with the higher baseline CRP levels (from 2.3 ± 0.3 mg/L to 1.7 ± 0.2 mg/L; *p* < 0.01). Mermigkis et al. [35] assessed the hs-CRP levels in 436 patients (252 males/184 females) with newly diagnosed moderate to severe OSAHS, before CPAP initiation and at the third and sixth month of the follow-up period. The results showed a delay in the normalization of CRP levels in females as compared to males, in spite of effective CPAP treatment. The minimum duration of CPAP therapy required to reduce CRP levels was 6 months in women, against only 3 months in men. Using as a basis, these findings that had well documented the significant reduction of hs-CRP levels in OSAHS patients with good long-term CPAP adherence, it had been suggested that good compliance to CPAP therapy may gradually reduce systemic inflammation, and thus improve disease-related cardiovascular morbidity [35]. Potential mechanisms that may explain the effect of CPAP therapy on systemic inflammation and the hs-CRP levels are suppression of apnea-induced nocturnal intermittent hypoxemia, normalization of renal sympathetic nerve activity, and improvement in sleep quality [14, 18].

Nonetheless, beneficial effects of long-term CPAP treatment on systemic inflammation in patients with OSAHS as assessed by hs-CRP levels are still unsettled and not supported by all studies. Huang et al. [36] assessed 78 OSAHS patients treated with CPAP therapy. After a follow-up of 12 months, a significant decrease of both ESS and AHI was observed, while the hs-CRP levels did not differ significantly as compared with baseline (from 2.71 ± 1.80 mg/L to 1.69 ± 1.40 mg/L; *p* = 0.429). Similarly, Karamanlı et al. [37] noted a nonsignificant decrease in the CRP levels (from 8.3 ± 8.5 mg/L to 6.2 ± 4.3 mg/L; *p* = 0.064) after a 3-month-CPAP therapy in 35 OSAHS patients. CPAP therapy may result in an increased upper airway inflammation. Indeed, Skoczyński et al. [38] reported a significant increase of the total number of inflammatory cells in the nasal lavage of OSAHS patients treated with CPAP, as compared to baseline values. This may be a consequence of the upper airway irritation caused by the delivered CPAP pressure.

### 4.3. Limitations of This Study

Five major limitations exist in the current study. First, a small sample was enrolled. Second, the assessment of OSAHS was based on a type 3 polygraph instead of a standardized polysomnographic recording, and no echocardiography study was performed. Third, controls could not be considered a standard matched control group, as they were recruited among patients referred for suspected SDB and were not reassessed for OSAHS after the 6-month-follow*-*up period. Fourth, untreated OSAHS patients were not included. Finally, as only patients without preexisting CVD were included, the effect of the nCPAP therapy on cardiac remodelling could not be assessed.

## 5. Conclusion

The major finding of our study is that long-term nCPAP application results in a significant reduction in serum levels of hs-CRP in compliant OSAHS patients without AHT, and this is following a dose-response relationship between the baseline serum levels of hs-CRP and the amplitude of its variation under nCPAP therapy. This result provides a further argument in favour of the potential cardiovascular protective role of CPAP therapy. It also suggests that hs-CRP would be useful as a valuable predictor in OSHAS treatment monitoring.

## Figures and Tables

**Table 1:** General characteristics of the four study groups.

	Groups				Total	*p*			
Variables/groups	G1 (*n* = 21)	G2 (*n* = 43)	G3 (*n* = 24)	G4 (*n* = 19)	*n* = 65	G1 vs. G2	G1 vs. G3	G1 vs. G4	G3 vs. G4
Mean age (years)	43.3 ± 10	45.98 ± 11	43.8 ± 12	48.6 ± 8.4	44.4 ± 10.8	NS	NS	NS	NS
Males/females	0.9	1.04	1.4	0.72	1	NS	NS	NS	NS
Smoking status *n* (%)	4 (19)	12 (27.9)	8 (33.3)	4 (21.1)	16 (25)	NS	NS	NS	NS
Alcoholism *n* (%)	1 (4.8)	3 (7)	3 (12.5)	0 (0)	4 (6.3)	NS	NS	NS	NS
ESS	7.7 ± 4.7	10.3 ± 3.2^∗^	9.5 ± 3.4	10.2 ± 2.9	9.4 ± 4	*0.013*	NS	NS	*0.015*
BMI (kg/m^2^)	32.9 ± 5.9	34.18 ± 6.5	32 ± 7.2	36.9 ± 4.3	33.78 ± 6.3	NS	NS	NS	*0.032*
CP (cm)	38.78 ± 3	41.75 ± 2.62	42 ± 2.68	41.5 ± 2.6	40.78 ± 3	*0.001*	*0.001*	*0.001*	NS
WHR	0.93 ± 0.07	0.98 ± 0.07	0.97 ± 0.06	0.99 ± 0.08	0.97 ± 0.07	*0.024*	NS	NS	NS
Mean SBP (mmHg)	125 ± 0.83	129.5 ± 1.23	120 ± 0.97	137 ± 1.16	128 ± 1.14	NS	NS	*<10^−4^*	*<10^−4^*
FVC (L)	3.59 ± 0.63	3.75 ± 0.77	3.72 ± 0.78	3.79 ± 0.79	3.70 ± 0.73	NS	NS	NS	NS
FEV1 (L)	3.00 ± 0.57	3.13 ± 0.63	3.12 ± 0.63	3.14 ± 0.65	3.09 ± 0.61	NS	NS	NS	NS
FEV1/FVC (%)	83.59 ± 5.69	83.25 ± 5.94	83.62 ± 5.40	82.781 ± 6.68	83.364 ± 5.82	NS	NS	NS	NS
AHI (events/h)	2.2 [0.9-4.8]	25.9 [7.1-78.9]	26 [8-77.9]	25.3 [7.1-78.9]	15.85 [0.9-78.9]	*<10^−3^*	*<10^−3^*	*<10^−3^*	NS
Mean SpO_2_ (%)	96.6 ± 1.4	92.82 ± 3.2	93.8 ± 2	91.6 ± 3.4	94.1 ± 3.3	*<10^−3^*	*<10^−3^*	*<10^−3^*	*0.034*
Min SpO_2_ (%)	92.3 ± 4.3	79.67 ± 9.4	81.5 ± 7	77.3 ± 11.2	83.8 ± 10.1	*<10^−3^*	*<10^−3^*	*<10^−3^*	NS
ODI (events/h)	1.8 [0-6.7]	20.2 [1-84.1]	15.85 [1-74.3]	23.2 [1.6-84.1]	7.15 [0-84.1]	*<10^−3^*	*<10^−3^*	*<10^−3^*	NS
TST90 (%)	0.0001 [0-3]	7.2 [0-82.3]	6.25 [0-27.6]	9.3 [0-82.3]	3.7 [0-82.3]	*<10^−3^*	*<10^−3^*	*<10^−3^*	NS
Serum glucose level (mmol/L)	5.21 ± 0.55	5.60 ± 0.70	5.5 ± 0.6	5.7 ± 0.8	5.5 ± 0.7	*0.029*	NS	NS	NS
HbA1c level (%)	5.57 ± 0.49	5.53 ± 0.47	5.7 ± 0.5	5.3 ± 0.34	5.5 ± 0.48	NS	NS	NS	NS
Serum urea level (mmol/l)	5.12 ± 1.1	5.27 ± 2.16	4.9 ± 1.5	5.8 ± 2.8	5.2 ± 1.9	NS	NS	NS	NS
Serum creatinine level (mmol/l)	62.2 ± 12.5	65.99 ± 20.35	66.3 ± 18.2	65.5 ± 23.3	64.7 ± 18.1	NS	NS	NS	NS
Creatinine clearance (ml/min)	116.9 ± 27	113.5 ± 34.2	113.9 ± 37.7	113 ± 32.7	114.6 ± 30.4	NS	NS	NS	NS

G1: controls. G2: patients with obstructive apnea-hypopnea syndrome (OAHS). G3: normotensive OAHS patients. G4: hypertensive OAHS patients. *n*: number. ESS: Epworth sleepiness scale. BMI: body mass index. CP: cervical perimeter. WHR: waist-hip ratio. SBP: systolic blood pressure. FVC: forced vital capacity. FEV1: forced expiratory volume in 1 second. AHI: apnea-hypopnea index. SpO_2_: transcutaneous oxygen saturation. ODI: oxygen desaturation index. TST90: total sleep time with SpO_2_ < 90%. HbA1c: glycated hemoglobin. Italic values indicate statistically significant difference.

**Table 2:** Distribution of OAHS patients and controls according to baseline hs-CRP levels.

Variables/groups	G1 *n* (%)	G2 *n* (%)	*p* G1 vs. G2
hs-CRP < mg/L	9 (42.9)	4 (9.3)	*0.003*
hs-CRP ≥ 1 mg/L	12 (57.1)	39 (90.7)	

OAHS: obstructive apnea-hypopnea syndrome. hs-CRP: high-sensitivity C-reactive protein. G1: OAHS patients. G2: controls. *n*: number.

**Table 3:** Predictive factors of high serum levels of hs-CRP in patients with OSAHS: univariate analysis.

Variables/groups	hs-CRP < 1 mg/L (*n* = 4)	hs-CRP ≥ 1 mg/L (*n* = 39)	*p*
Males/females	2/2	20/19	0.99
Age (years)	39.25 ± 6.85	46.67 ± 11.23	0.20
Smoking status *n* (%)	1 (25)	11 (28.2)	0.89
AHT *n* (%)	1 (25)	18 (46.2)	0.61
ESS	7.25 ± 3.30	10.62 ± 3.10	0.053
BMI (kg/m^2^)	30.86 ± 4.32	34.52 ± 6.64	0.29
AHI (events/h)	12.30 [8.10-21.30]	26 [7.10-78.9]	*0.021*
Mean SpO_2_ (%)	93.70 ± 2.82	92.44 ± 3.33	0.30
Min SpO_2_	82.76 ± 7.54	78.34 ± 10.00	0.08
ODI (events/h)	9.15 [2.30-20.20]	23.20 [1-84.10]	0.09
TST90 (%)	9.90 [2.40-20]	7.14 [0-82.3]	0.78
FVC (L)	3.66 ± 0.55	3.76 ± 0.80	0.80
FEV1 (L)	3.07 ± 0.43	3.14 ± 0.65	0.85
FEV1/FVC	84.17 ± 5.15	83.15 ± 6.07	0.74
Blood glucose level (mmol/L)	5.15 ± 0.28	5.65 ± 0.71	0.17
Creatinine clearance (ml/min)	152.75 ± 24.47	126.71 ± 35.72	0.16

hs-CRP: high-sensitivity C-reactive protein. OAHS: obstructive apnea-hypopnea syndrome. *n*: number. AHT: arterial hypertension. ESS: Epworth sleepiness score. BMI: body mass index. AHI: apnea-hypopnea index. SpO_2_: transcutaneous oxygen saturation. ODI: oxygen desaturation index. TST90: total sleep time with SpO_2_ < 90%. FVC: forced vital capacity. FEV1: forced expiratory volume in 1 second. Italic values indicate statistically significant difference.

## Data Availability

Data are available on request through corresponding author (sameh MSAAD, pneumo1972@gmail.com).
